# Feasibility and first reports of the MATCH-R repeated biopsy trial at Gustave Roussy

**DOI:** 10.1038/s41698-020-00130-7

**Published:** 2020-09-08

**Authors:** Gonzalo Recondo, Linda Mahjoubi, Aline Maillard, Yohann Loriot, Ludovic Bigot, Francesco Facchinetti, Rastislav Bahleda, Anas Gazzah, Antoine Hollebecque, Laura Mezquita, David Planchard, Charles Naltet, Pernelle Lavaud, Ludovic Lacroix, Catherine Richon, Aurelie Abou Lovergne, Thierry De Baere, Lambros Tselikas, Olivier Deas, Claudio Nicotra, Maud Ngo-Camus, Rosa L. Frias, Eric Solary, Eric Angevin, Alexander Eggermont, Ken A. Olaussen, Gilles Vassal, Stefan Michiels, Fabrice Andre, Jean-Yves Scoazec, Christophe Massard, Jean-Charles Soria, Benjamin Besse, Luc Friboulet

**Affiliations:** 1 Université Paris-Saclay, Institut Gustave Roussy, Inserm U981, Biomarqueurs prédictifs et nouvelles stratégies thérapeutiques en oncologie, 94800 Villejuif, France; 2 Drug Development Department (DITEP), Gustave Roussy Cancer Campus, Villejuif, France; 3 Department of biostatistics and epidemiology, Gustave Roussy Cancer Campus, Villejuif, France; 4 Department of Medical Oncology, Gustave Roussy Cancer Campus, Villejuif, France; 5 Experimental and Translational Pathology Platform (PETRA), Genomic Platform - Molecular Biopathology unit (BMO) and Biological Resource Center, AMMICA, INSERM US23/CNRS UMS3655, Gustave Roussy Cancer Campus, Université Paris-Saclay, Villejuif, France; 6 Department of Medical Biology and Pathology, Gustave Roussy Cancer Campus, Villejuif, France; 7 Department of Clinical Research, Gustave Roussy Cancer Campus, Université Paris-Saclay, Villejuif, France; 8 Department of Interventional Radiology, Gustave Roussy Cancer Campus, Villejuif, France; 9 XenTech, Evry, France; 10 Department of Hematology, Gustave Roussy Cancer Campus, Villejuif, France

**Keywords:** Biomarkers, Cancer genetics

## Abstract

Unravelling the biological processes driving tumour resistance is necessary to support the development of innovative treatment strategies. We report the design and feasibility of the MATCH-R prospective trial led by Gustave Roussy with the primary objective of characterizing the molecular mechanisms of resistance to cancer treatments. The primary clinical endpoints consist of analyzing the type and frequency of molecular alterations in resistant tumours and compare these to samples prior to treatment. Patients experiencing disease progression after an initial partial response or stable disease for at least 24 weeks underwent a tumour biopsy guided by CT or ultrasound. Molecular profiling of tumours was performed using whole exome sequencing, RNA sequencing and panel sequencing. At data cut-off for feasibility analysis, out of 333 inclusions, tumour biopsies were obtained in 303 cases (91%). From these biopsies, 278 (83%) had sufficient quality for analysis by high-throughput next generation sequencing (NGS). All 278 samples underwent targeted NGS, 215 (70.9%) RNA sequencing and 222 (73.2%) whole exome sequencing. In total, 163 tumours were implanted in NOD scid gamma (NSG) or nude mice and 54 patient-derived xenograft (PDX) models were established, with a success rate of 33%. Adverse events secondary to invasive tumour sampling occurred in 24 patients (7.6%). Study recruitment is still ongoing. Systematic molecular profiling of tumours and the development of patient-derived models of acquired resistance to targeted agents and immunotherapy is feasible and can drive the selection of the next therapeutic strategy.

## Introduction

Cancer research has led to significant advances in the understanding of tumour biology and immunology, providing a rational for the development of novel treatment strategies^1^. In part, this has been feasible due to the improved access of high throughput molecular biology techniques, the improvements in developing patient-derived models and the collaborative efforts of the research community to comprehensively characterize cancer biology^2,3^.

In recent years, the breakthrough of highly effective treatments such as immune checkpoint inhibitors and molecular targeted therapies has improved outcomes for patients affected by different types of cancer and radically changed their clinical management^4,5^. Many innovative approaches, using anti PD1/PDL1 checkpoint inhibitors, kinase inhibitors, antibody-drug conjugates, monoclonal antibodies, cell-cycle inhibitors, endocrine therapies, DNA repair and epigenetic modulators have become standard therapeutic options for selected cancer patients^6^. This vast landscape of drugs in development, used either as monotherapy or in combination, will continue to improve cancer care in the near future^6–10^.

In this context, the development of reliable biomarkers is key to predict clinical benefit of therapies and avoid unnecessary toxicities. For instance, targetable molecular alterations in *EGFR*, *BRAF*, *MET*, *RET, ROS1, ALK, NTRK, KIT* predict responses to specific kinase inhibitors^11–18^. PD-L1 staining, tumour mutational burden, T-effector signatures and mutational signatures are currently being studied as predictive biomarkers of treatment with immune checkpoint inhibitors^19^.

However, even when prolonged disease control can be achieved, disease progression, secondary to acquired resistance to antineoplastic treatments, eventually occur. Multiple resistance mechanisms to targeted therapies have been characterized, shedding light on the evolution of cancer cells under treatment pressure^20^. This has subsequently guided the development of novel compounds capable of overcoming these barriers to provide patients with new therapeutic alternatives^21,22^.

As new treatments are being developed, cancer cells will consequently adapt to sustained tumour proliferation and dissemination^23^. Hence, it is crucial to design research strategies intended to systematically study novel resistance mechanisms to cancer therapies.

Herein, we report the study design and feasibility of the MATCH-R study, a prospective single institution trial, designed to identify mechanisms of acquired resistance in patients with advanced cancer treated with molecular targeted agents and immunotherapy.

## Results

### Study population

**Fig. 1:**
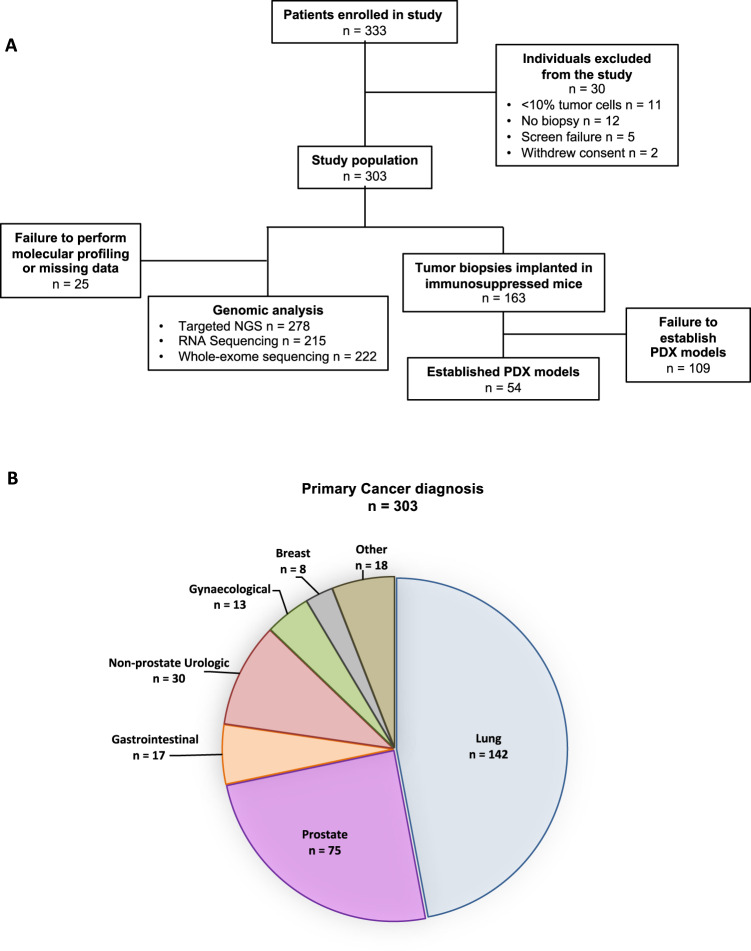
MATCH-R patient inclusions. **a** Study flowchart. **b** Proportion of histological types included in the study.

From January 1st 2015 and as of June 15th, 2018, a total of 333 inclusions were recorded (Fig. 1a). Thirty cases (9%) were later excluded from the analysis due to screen failure (*n* = 5), withdrawal of consent (*n* = 2), absence of tumour biopsy (*n* = 12) and inadequate tumour content in the biopsy for molecular analysis (*n* = 11) (Fig. 1a). From the 303 biopsies with adequate tumour content (tumour cellularity ≥ 10%), 159 (52.5%) were included in cohort 1 (Global Match-R), 12 (4%) in cohort 2 (NSCLC EGFR + /ALK+), 57 (18.8%) in cohort 3 (Immunotherapy) and 75 (24.8%) in cohort 4 (Prostate cancer) (Supplementary Table 1). The study is currently open to enrolment.

At this interim cut-off, median age (interquartile range) for the study population was 65 years (55–71) with a higher proportion of men (60.1%) (Supplementary Table 1). The most common cancer types were non-small cell lung cancer (NSCLC) (*n* = 142) followed by prostate (*n* = 75), urothelial (*n* = 30), gastrointestinal (*n* = 17), gynaecological (*n* = 13) and breast cancers (*n* = 8). Patients with less frequent tumour types were also included (Fig. 1b).

**Fig. 2:**
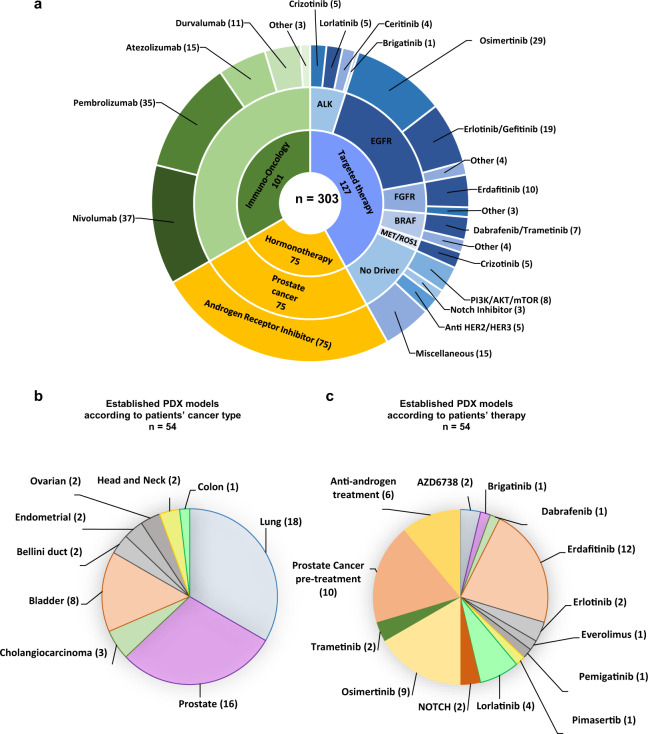
Drivers, treatments and models. **a** Distribution of molecular drivers and anticancer treatments of patients included in MATCH-R. In blue, molecular targeting agents; in green, immuno-oncology related treatments; in orange, Androgen Receptor inhibitor for prostate cancer. **b** PDX and/or cell lines models developed grouped by histological sub-types. **c** PDX and/or cell lines models developed grouped by treatments received.

Regarding the last cancer treatment received at the time of inclusion, 127 patients (42%) had experienced disease progression with targeted therapies, 101 (33%) with immunotherapy and 75 (25%) with anti-androgen therapy (Fig. 2a).

### Feasibility of tumour biopsies

Overall, the mean tumour content of all 303 biopsies that underwent NGS was 49%. In most cases, the procedure was safe and well tolerated, and procedure-related adverse events were reported in 24 patients among 314 patients who underwent a biopsy (7.6%), of which the most common were the development of pneumothorax (14) and bleeding (2) (Table 1), which seems consistent with previous studies^24^. Of note, a concomitant double biopsy of progressive and stable lesions were performed in 21 patients. Twelve patients did not undergo tumour biopsy, which was due to technical or clinical factors such as lack of accessible tumour sites, renal insufficiency, previous pneumothorax and anxiety.

**Table 1:** Adverse events (AE) related to biopsy procedure among 24 patients with at least one AE.

Adverse events		*N*
Bleeding		2
Pneumothorax	All grades	14
	Grade 1	5
	Grade 2	1
	Grade 3	7
	Missing grade	1
Other		10

### Feasibility of molecular analysis

From the 303 biopsies with ≥10% tumour cells that underwent NGS, results were obtained for 278 samples (92%) (Fig. 1a). Of these, 222 samples were analyzed with WES and 215 with RNA sequencing. Importantly, 197 samples (65%) were fully characterized by targeted NGS, WES and RNA sequencing. These preliminary feasibility results show that systematic molecular profiling of tumours acquiring resistance to specific anticancer therapies is achievable.

### Establishment of patient-derived models of resistance

Until the interim cut-off, 163 tumours biopsies were implanted in immune-deficient mice (Supplementary Table 2). The success rate for the development of PDX models reached 33%, being the highest for bladder urothelial carcinomas (72.7%). The most frequently implanted tumours derived from patients with NSCLC (*n* = 59) and castrate-resistant prostate cancer (*n* = 60) with success rates of 30% and 27%, respectively. Among the 54 established PDX models, 12 were developed from FGFR-driven tumours resistant to erdafitinib, 9 from EGFR-mutant osimertinib resistant tumours and 4 from ALK-rearranged lung cancers resistant to lorlatinib (Fig. 2b, c). Considering the prostate PDX models, 10 were established from biopsies prior to anti-androgen receptor treatments and 6 from anti-androgen receptor resistant tumours, and in one case, paired pre and post-treatment PDX models were developed. The remaining established PDX models were derived from patients treated with ATR, NOTCH, MEK or BRAF inhibitors.

The average time from tumour implantation to the first passage in mice was approximately 3 months. Although PDX models were essential to deepen the characterization of resistance mechanisms and to identify overcoming strategies, the data generated on the PDX models were usually not timely enough to be used in the clinical decision making. Of note, only few cell lines were obtained directly from patient biopsies whereas stable growth in vitro was systematically obtained when dissociating tumour cells from the PDX. This is most probably linked to the quantity (number of tumour cells) and quality (percentage of tumour cells) of the PDX samples compared to corresponding patient biopsies.

Importantly, when applying a selective pressure using the same drug that the patient had experience before disease progression, 11 out of 12 PDX models tested by XenTech recapitulated the pharmacological response observed in patients, both from progression and stable sites (Supplementary Fig. 2). This suggests that the timely application of selective pressure in vivo allowed to maintain the resistance mechanisms that were acquired in patients. It is noteworthy that sequencing of the PDX closely recapitulated the molecular profile of the patient biopsies, which systematically showed persistence of initial oncogenic driver alteration.

### Molecular characterization and clinical implication of the results

Among 127 patients having received a targeted therapy, the alterations in driver oncogenes detected were mainly found in ALK (15), EGFR (52), FGFR (13) and BRAF (11) (Fig. 2a). In addition to the driver oncogene detection, the sequencing results shed light on potential acquired resistance mechanisms. Among those 127 patients, 57 (45%) acquired a targetable alteration at resistance. Thirty-two (25%) patients acquired a secondary mutation within the targeted oncogene (on-target resistance mechanisms) such as T790M after 1st generation EGFR tyrosine kinase inhibitor (TKI), or C797S when post-osimertinib. Twenty-five (20%) patients acquired a mutation (or a translocation) in a gene involved in kinase signalling such as MEK, TSC1, PTEN, NF1, RAS, PIK3CA bypassing the inhibition of the original driver oncogene (off-target resistance mechanisms). If the drug is available, patients with an on-target resistance mechanism could benefit from a treatment with a next generation TKI, whereas patients with an off-target resistance mechanisms could benefit from a combination treatment targeting the original driver oncogene and the activated bypass. In our study, among 57 patients with an acquired targetable alteration, 26 (46%) received an adjusted treatment aiming at overcoming the identified alteration causing resistance. These numbers are noteworthy high taking into account the limitations related to actual drug availability and/or drug combination approvals. Altogether, these results revealed that performing a biopsy and high-throughput sequencing at the time of relapse to targeted therapy can allow the selection of a next precision medicine strategy for at least 20% of patients who relapse on a targeted therapy (26/127).

In the context of the MATCH-R trial, we have recently reported multiple resistance mechanisms to ALK, ROS1 and EGFR TKIs. For instance, we characterized diverse resistance mechanisms occurring in patients with ALK-rearranged NSCLC progressing on the third-generation ALK inhibitor lorlatinib such as additional ALK secondary compound mutations, and NF2 loss of function mutations^25^. In addition, we described the ROS1 S1986Y/F kinase domain resistance mutations in a patient with ROS1-rearranged lung cancer progressing on crizotinib^26^. Similarly, multiple oncogenic fusions involving FGFR3, RET or ALK were identified as common resistance mechanisms to the third-generation EGFR TKI osimertinib in EGFR-mutant NSCLC patients^27^. Lastly, we identified MEK1, NRAS, PTEN and KRAS mutations among BRAFV600E NSCLC patients progressing on dabrafenib-trametinib combination^28^.

## Discussion

Systematic molecular profiling of tumours has been proposed as a diagnostic tool to tailor treatment according to the patient’s cancer genotype and phenotype. Previous prospective studies investigated the feasibility of integrating real-time molecular findings for individualized treatment^29–35^. The BATTLE trial was the first, biopsy mandated, biomarker-based study that demonstrated the feasibility of selecting personalized treatment based on molecular biomarkers revealing an 8-week disease control rate in 46% of chemoresistant NSCLC patients^35^. In the MOSCATO-01 trial, 33% of heavily pre-treated patients achieved a clinical benefit to targeted therapies, which were assigned based on tumour molecular profiling^32^. More recently, the WINTHER trial revealed a disease control rate (DCR) of 26.2% when DNA sequencing or RNA expression were used to determine the therapy given to colon, head and neck, and lung cancer patients^34^. Even more importantly, the I-PREDICT study investigated the feasibility of the selection of a customized multidrug regimen when multiple molecular alterations were identified by DNA sequencing. The authors validated the feasibility of this approach and were able to administer ≥1 matched drug to 49% of patients^33^. These studies promoted the use of molecular biomarkers to select patients for optimal treatment assignments and revealed that genomic and transcriptomic profiling are both useful for improving personalized cancer treatment recommendations and patient outcome.

The MATCH-R trial is another step forward showing the clinical usefulness of high-throughput genomic and transcriptomic profiling of repeated biopsies in the context of acquired resistance to anticancer agents. Virtually, every patient will eventually acquire a resistance mechanism despite a personalized treatment, and selecting the next treatment regimen in such a context of heterogeneous or adaptive disease remains challenging. The MATCH-R study in addition to providing new insights on acquired resistance mechanisms to a variety of antineoplastic treatments, in a wide range of cancer types, aims at prolonging the patients benefit by readapting the treatment at resistance according to a repeated molecular profiling of patient tumours. Our feasibility results show that 92% of tumour samples with ≥10% tumour cells are suitable for molecular analysis, with complete molecular profiling achievable in 65% of cases. The information obtained from this study is integrated in the clinical context of the patient and discussed in a dedicated molecular tumour board who designs tailored therapeutic options in the setting of resistance. Although the number of patients oriented towards a novel treatment can be limited by drug inaccessibility (lack of potent inhibitor, no clinical trial available or drug combinations not previously evaluated in patients), 46% (26/57) of patients presenting with an acquired targetable alteration received an adjusted treatment, which is a similar number to what was observed in the I-PREDICT study. Prolonging the clinical benefit for these metastatic cancer patients will remain important and our best option to maximize the clinical outcomes and justifies a costly repeat biopsy program.

When feasible, patient-derived in vivo and in vitro models of resistance are developed to further characterize mechanisms of resistance. This collection of PDX/cell line models remains a useful preclinical tool to identify pivotal mechanisms underlying acquired resistance to current targeted therapies and will allow to develop innovative strategies to overcome or prevent treatment failure.

We conclude that a systematic and longitudinal study of mechanisms of resistance to targeted therapies and immunotherapy by molecular profiling and the development of patient-derived models is feasible, safe and contributes to clinical benefits for metastatic cancer patients.

## Methods

### Study design and eligibility criteria

The MATCH-R trial (NCT02517892) is an ongoing prospective, single institution study held at Gustave Roussy Cancer Campus. The primary objective of this study is to characterize molecular mechanisms of acquired resistance to targeted therapies and immunotherapy in patients with advanced cancer by NGS and the development of patient derived xenografts (PDX) and cell lines. Patients must have had either an initial response, defined as partial response (PR) or complete response (CR) by RECIST 1.1, or a stable response (SD) for at least 24 weeks, and develop disease progression while actively receiving molecular targeted therapy or immunotherapy. Key eligibility criteria for study entry are summarized in Table 2.

**Table 2:** Key inclusion and exclusion criteria.

*Inclusion criteria*	
	Unresectable or metastatic cancer diagnosis
	Treatment with selected targeted agents or immunotherapy
	Disease progression while actively on treatment after achieving an initial response to treatment (defined as a partial or complete response by RECIST 1.1 or stable disease lasting longer than 24 weeks)
	Progressing tumour lesion accessible to core biopsies, including malignant pleural effusion and ascites
	The interval of time between the last dose of the selected therapy and the tumour biopsy should be less or equal to one month
	Availability of tumour tissue acquired before the initiation of the selected therapy
	Fully informed, able to comply with the protocol and signed the informed consent
*Exclusion criteria*	
	Clinical contraindications to biopsy procedure (coagulation abnormalities)

**Fig. 3:**
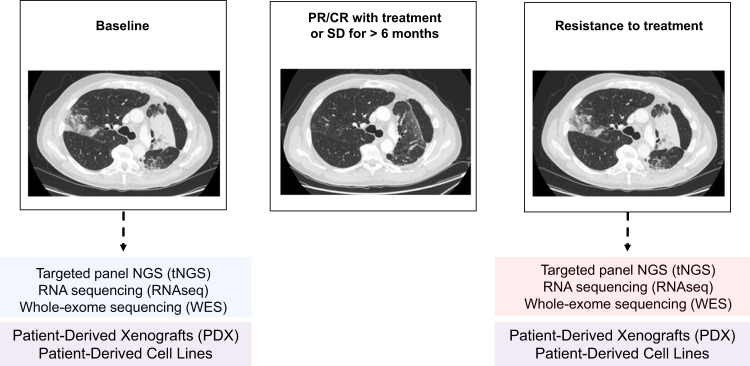
MATCH-R study design. Tumour biopsies are obtained at treatment resistance and at treatment baseline. Tumour samples undergo deep molecular analysis and some are selected to develop patient derived xenografts.

Baseline or pre-treatment samples are obtained either from diagnostic formalin fixed paraffin embedded (FFPE) pathology blocks or from fresh biopsies if available. Post-progression tumour samples are obtained by core biopsies stored as frozen samples and embedded in paraffin (Fig. 3), as well as from serous effusions. If considered safe, concomitant target lesions with stable disease are biopsied and analyzed to compare genetic alterations driving disease progression in sub-clonal populations. The target lesions undergo several biopsies to provide adequate material for pathological diagnosis, complete molecular profiling and to develop patient-derived models. In addition, blood samples are collected longitudinally throughout the treatment and at progression in selected patients for sequencing of circulating tumour DNA (ctDNA).

The expected events for the primary objective are the detection of new molecular alterations, the disappearance of pre-existing alterations, and significant changes in the allele frequencies or the proportion of cells with molecular alterations at the time of resistance. The aim is to identify genes that are altered in more than 10% of the patients who develop resistance to a given drug. Genes for which a pathogenic event is found in at least 2 patients will be selected for further functional laboratory studies, and we plan to study 52 patients per type of molecular targeting agents. With this sample size, the probability to miss a gene that is really altered in more than 10% of the patients who develop resistance is less than 20% (power > 80%).

The study was amended from its original design, that required only a post-treatment biopsy (which became cohort 1), to include specific cohorts of patients with paired pre- and post-treatment biopsies (cohorts 2–4). This aimed to increase the precision of this study in the assessment of acquired mechanisms of resistance of anticancer drugs. These cohorts include: patients treated with EGFR/ALK inhibitors in oncogene driven non-small cell lung cancer (NSCLC EGFR+/ALK+) (cohort 2), patients treated with immunotherapy for lung cancer and bladder cancer (cohort 3) and patients with prostate cancer resistant to androgen deprivation therapy (ADT) (cohort 4). Unfortunately the amendment was approved lately and only few patients benefited yet from pre- and post-treatment biopsies (7 in the EGFR/ALK cohort and 13 in the immunotherapy cohort).

### Circuit and logistic of samples

Patients are included during a consultation with their referring physician and sign the informed consent (Supplementary Fig. 1). A dedicated physician checks that the inclusion criteria are respected and medical assistants set a biopsy date and inform the other services involved. Interventional radiologists validate the feasibility of the biopsy procedure. On the day of the biopsy, interventional radiologists collect several tumour samples guided by CT or ultrasound. The biopsies are immediately delivered to the sample management service where they are either fixed, frozen or kept fresh. Fresh biopsies are directly implanted in the sub-renal capsules of immunocompromised mice, fixed biopsies are included in paraffin, and frozen biopsies in optimal cutting temperature (OCT) in the histopathology department. The percentage of tumour cells is determined by H&E stain on three frozen biopsies and the biopsy with the highest percentage is brought to the molecular biology department for DNA and RNA extraction for NGS. After about 3–6 weeks of turnaround time, the molecular biology department establishes a report of sequencing results in order to be discussed in the molecular tumour board. All clinical, histological and molecular data are recorded in the case report form (CRF).

### Molecular analyses

Tumour biopsies are evaluated by senior pathologists to estimate the percentage of tumour cells, using a threshold of 10% tumour cells in order to perform the molecular analysis. Targeted NGS is performed with the Ion Torrent PGM (ThermoFisher Scientific) sequencer using a customized panel (Mosc4) covering 82 cancer genes developed with Ion AmpliSeq custom design. The bioinformatics analysis is performed using TorrentSuite software, variantCaller (ThermoFisher Scientific). If the proportion of tumour cells is higher than 30%, whole exome sequencing (WES), and RNA sequencing (RNAseq) are also performed in a clinical-grade laboratory. For WES, the mean coverage was 140X.

### Molecular tumour board

On a weekly basis a group of 10 people composed of clinicians, biologists and scientists review the sequencing results and generate a report. Targeted NGS results are usually discussed first but WES and RNA sequencing data are also incorporated into the discussions as soon as they are available, in order to confirm or complete the molecular profile. For every patient included in the MATCH-R trial, the ESMO Scale for *Clinical Actionability of molecular Targets* (ESCAT) and the OncoKB databases are used to rank genomic alterations as potential targets for cancer precision medicine and to predict the effects and treatment implications of specific cancer gene alterations. According to the availability of registered drugs or ongoing clinical trials, patients are oriented towards adjusted treatment strategies whenever possible.

### Establishment of patient derived models

Fresh tumour fragments are implanted in the sub-renal capsule of NOD scid gamma (NSG) or nude mice obtained from Charles River Laboratories. Xenografts are then serially propagated subcutaneously from mice to mice up to five passages to generate a viable tumour bank. From passage 3, selective pressure with the inhibitor to which the patient acquired resistance is applied to the mice, to avoid expansion of sensitive tumour cell populations. This is performed through a collaboration with the PDX-dedicated CRO (XenTech).

Patient-derived cell lines are developed from (a) patient biopsies or (b) PDX samples. (a) Patient biopsies are cut in petri dishes and incubated with Liberase™ DH Research Grade (Ref 5401054001, Sigma Aldrich) at 37 °C for 1 h; (b) PDX samples are processed by enzymatic digestion with the tumour dissociation kit (Ref.130-095-929, Miltenyi Biotec) and mechanic degradation with the gentleMACs^TM^ dissociator. Cells are cultured with DMEM/F-12+GlutamMAX^TM^ 10% FBS and 10% enriched with hydrocortisone 0.4 µg/ml, cholera toxin 8.4 ng/ml, adenine 24 µg/ml and ROCK inhibitor 5 µM (Y-27632, S1049 Selleckchem) until a stable proliferation of tumour cells is observed.

### Ethics

All patients participating in the study are fully informed and sign an informed consent. The MATCH-R trial has been approved by the ethics committee at Institut Gustave Roussy, the French National Agency for Medicines and Health Products Safety (ANSM), and is conducted in accordance with the Declaration of Helsinki. Demographic and clinical data are prospectively collected together with pathology records and integrated with molecular analysis and translational research studies. All animal procedures and studies have been approved by the French *Ministère de ‘Enseignement supérieur, de la Recherche et de l’Innovation* (APAFIS#2790-2015112015055793).

### Reporting summary

Further information on research design is available in the Nature Research Reporting Summary linked to this article.

## Supplementary information

Supplementary Information

Reporting Summary

## Data Availability

Data from this clinical trial are available from the authors and can be requested by filling out the data request form for Gustave Roussy clinical trials at https://redcap.gustaveroussy.fr/redcap/surveys/?s=DYDTLPE4AM. The trial steering committee and the sponsor will review the requests on a case-by-case basis. In case of approval, a specific agreement between the sponsor and the researcher may be required for data transfer.
