# Supplementary material for: Feasibility and first reports of the MATCH-R repeated biopsy trial at Gustave Roussy
Source: NPJ Precis Oncol. 2020 Sep 8;4:27. doi: 10.1038/s41698-020-00130-7 (PMC7478969; doi:10.1038/s41698-020-00130-7)

Supplementary Table 1. Patients characteristics (n=303)

Patients characteristics	n	(%)
**Age**		
Median (IQR)	65 (55-71)	
**Gender**		
Female	121	39.9
Male	182	60.1
**Cohorts**		
1- MATCH-R Global	159	52.5
2- EGFR/ALK	12	4.0
3- Immunotherapy	57	18.8
4- Prostate Cancer	75	24.8

Supplementary Table 2. Feasibility of the development of patient-derived xenograft models per cancer type.

Cancer Type	Tumor implanted (n)	PDX models developed (n)	Success rate (%)
Lung	59	18	30.5
Prostate	60	16	26.7
Cholangiocarcinoma	15	3	20
Bladder	11	8	72.7
Bellini Tumor	3	2	66.7
Endometrial	4	2	50
Ovarian	3	2	66.7
Head and Neck	3	2	66.7

Colon	3	1	33.3
Adenoid Cystic Carcinoma	1	0	0
**Total**	**163**	**54**	**33.1**

Supplementary Figure 1

Circuit and logistics of sample and treatment tailoring

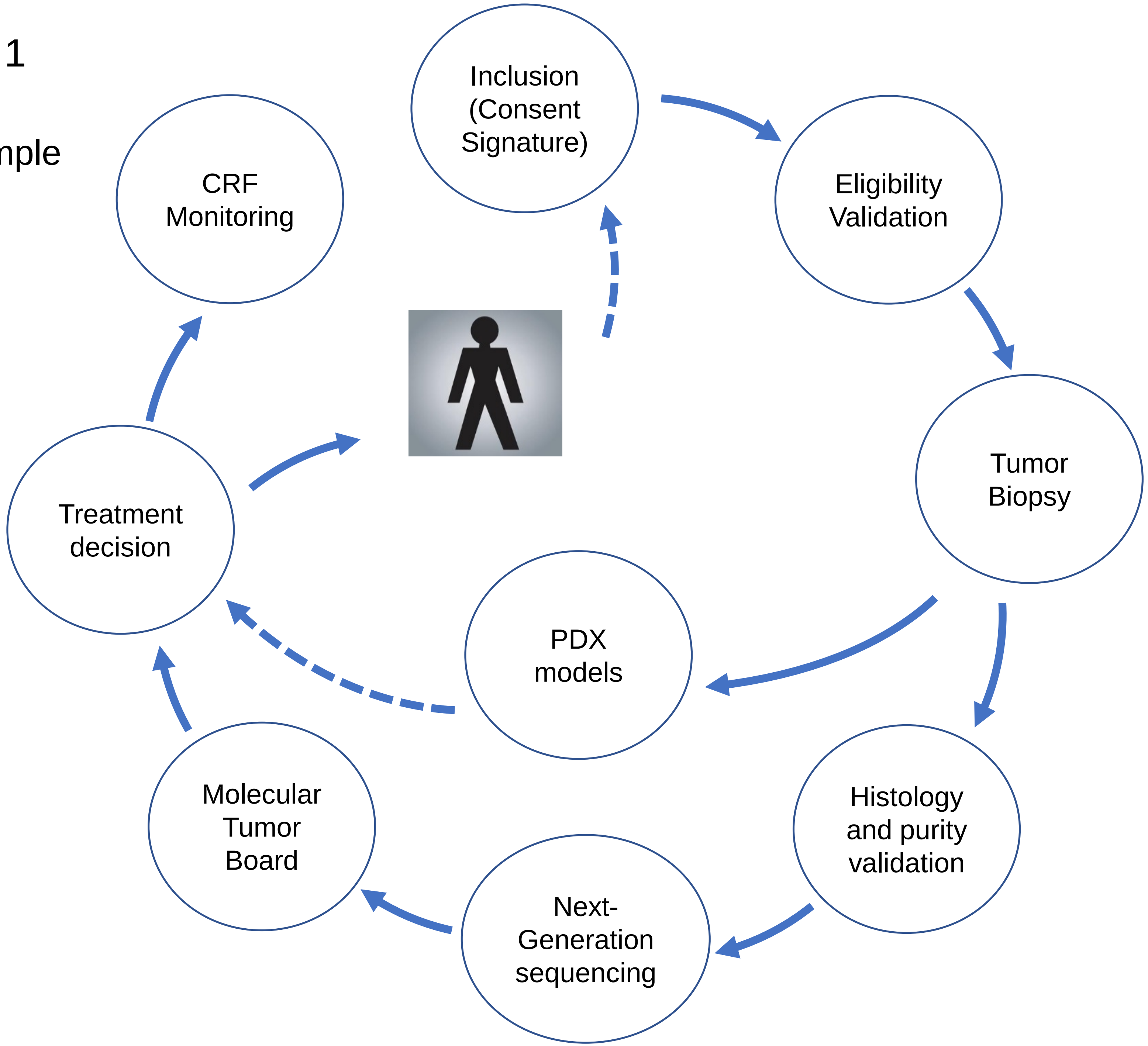

Supplementary Figure 2

Example of PDX growth, with or without drug treatment, established from stable disease site (SD) or progressive disease site (PD). MR15 was a patient with FGFR3-S249C driven urothelial cancer with acquired resistance to erdafitinib.

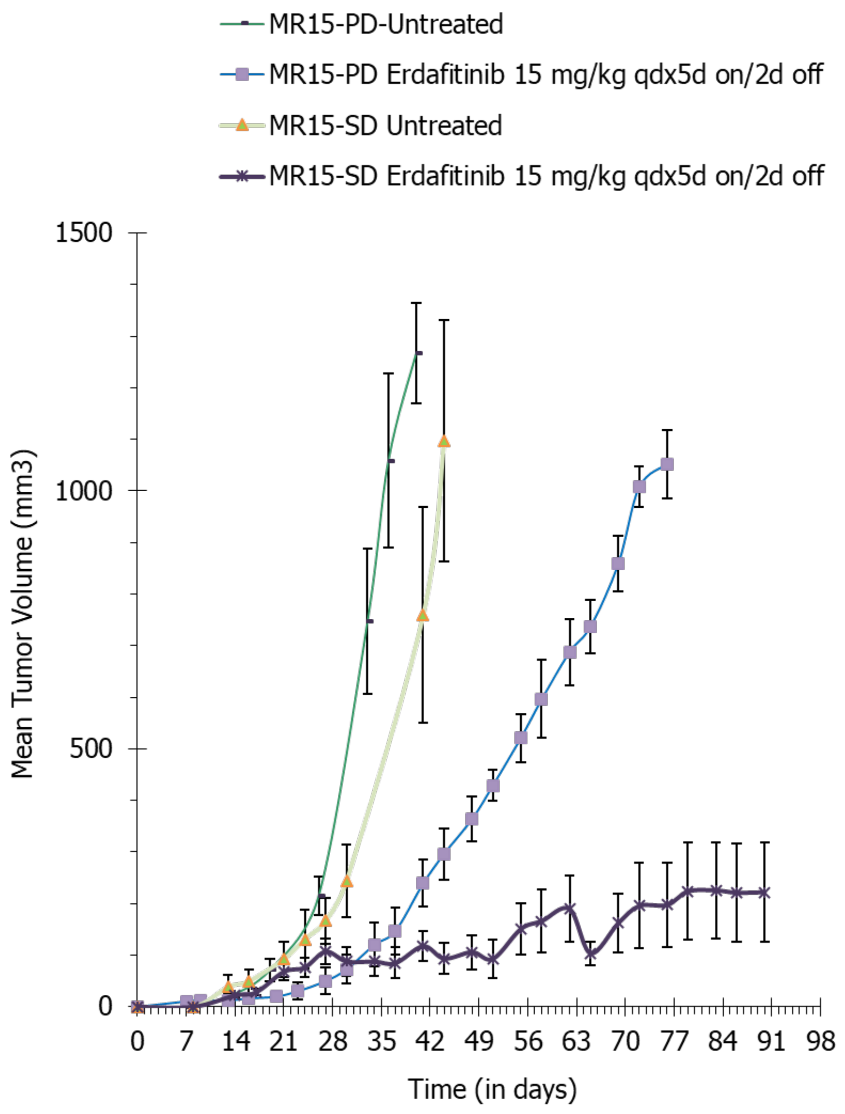

Supplement: Supplementary file 1 — Supplementary Information [file 41698_2020_130_MOESM1_ESM.pdf]
