# Supplementary material for: Feasibility and first reports of the MATCH-R repeated biopsy trial at Gustave Roussy
Source: NPJ Precis Oncol. 2020 Sep 8;4:27. doi: 10.1038/s41698-020-00130-7 (PMC7478969; doi:10.1038/s41698-020-00130-7)
Supplement: Supplementary file 2 — Reporting Summary [file 41698_2020_130_MOESM2_ESM.pdf]

nature research

Corresponding author(s): NPJPRECISIONONCOLOGY-00382R

Last updated by author(s): Jul 9, 2020

Reporting Summary

Nature Research wishes to improve the reproducibility of the work that we publish. This form provides structure for consistency and transparency in reporting. For further information on Nature Research policies, see Authors & Referees and the Editorial Policy Checklist.

Statistics

For all statistical analyses, confirm that the following items are present in the figure legend, table legend, main text, or Methods section.

n/a	Confirmed	
☐	☒	The exact sample size (n) for each experimental group/condition, given as a discrete number and unit of measurement
☒	☐	A statement on whether measurements were taken from distinct samples or whether the same sample was measured repeatedly
☒	☐	The statistical test(s) used AND whether they are one- or two-sided<br>*Only common tests should be described solely by name; describe more complex techniques in the Methods section.*
☒	☐	A description of all covariates tested
☒	☐	A description of any assumptions or corrections, such as tests of normality and adjustment for multiple comparisons
☒	☐	A full description of the statistical parameters including central tendency (e.g. means) or other basic estimates (e.g. regression coefficient) AND variation (e.g. standard deviation) or associated estimates of uncertainty (e.g. confidence intervals)
☒	☐	For null hypothesis testing, the test statistic (e.g. F, t, r) with confidence intervals, effect sizes, degrees of freedom and P value noted<br>*Give P values as exact values whenever suitable.*
☒	☐	For Bayesian analysis, information on the choice of priors and Markov chain Monte Carlo settings
☒	☐	For hierarchical and complex designs, identification of the appropriate level for tests and full reporting of outcomes
☒	☐	Estimates of effect sizes (e.g. Cohen's d, Pearson's r), indicating how they were calculated

Our web collection on statistics for biologists contains articles on many of the points above.

Software and code

Policy information about availability of computer code

Data collection	Data was collected using an electronic CRF with Macro software
Data analysis	The data analysis for this paper was generated using SAS software v9.4

For manuscripts utilizing custom algorithms or software that are central to the research but not yet described in published literature, software must be made available to editors/reviewers. We strongly encourage code deposition in a community repository (e.g. GitHub). See the Nature Research guidelines for submitting code & software for further information.

Data

Policy information about availability of data

All manuscripts must include a data availability statement. This statement should provide the following information, where applicable:

- Accession codes, unique identifiers, or web links for publicly available datasets
- A list of figures that have associated raw data
- A description of any restrictions on data availability

Data from this clinical trial are available from the authors and can be requested by filling out the data request form for Gustave Roussy clinical trials at https://redcap.gustaveroussy.fr/redcap/surveys/?s=DYDTLPE4AM.
The trial steering committee and the sponsor will review the requests on a case-by-case basis. In case of approval, a specific agreement between the sponsor and the researcher may be required for data transfer.

Field-specific reporting

Please select the one below that is the best fit for your research. If you are not sure, read the appropriate sections before making your selection.

☒ Life sciences ☐ Behavioural & social sciences ☐ Ecological, evolutionary & environmental sciences

For a reference copy of the document with all sections, see nature.com/documents/nr-reporting-summary-flat.pdf

Life sciences study design

All studies must disclose on these points even when the disclosure is negative.

Sample size	The events will be grouped by gene at the patient level. The objective is to identify genes that are altered in more than 10% of the patients who develop resistance to treatments. Genes for which a pathogenic event is found in at least 2 patients will be selected, and we plan to study 52 patients per group of molecular targeting agents. With this sample size, the probability to miss a gene that is really altered in more than 10% of the patients who develop resistance is less than 20% (power > 80%)
Data exclusions	No data exclusions
Replication	No experimental findings are included in this study
Randomization	This is not relevant to our study as this is not a randomized trial
Blinding	This is not relevant to our study as no group allocation was required in our study

Reporting for specific materials, systems and methods

We require information from authors about some types of materials, experimental systems and methods used in many studies. Here, indicate whether each material, system or method listed is relevant to your study. If you are not sure if a list item applies to your research, read the appropriate section before selecting a response.

Materials & experimental systems

n/a	Involved in the study	
☒	☐	Antibodies
☒	☐	Eukaryotic cell lines
☒	☐	Palaeontology
☐	☒	Animals and other organisms
☐	☒	Human research participants
☐	☒	Clinical data

Methods

n/a	Involved in the study	
☒	☐	ChIP-seq
☒	☐	Flow cytometry
☒	☐	MRI-based neuroimaging

Animals and other organisms

Policy information about studies involving animals; ARRIVE guidelines recommended for reporting animal research

Laboratory animals	3 weeks old NSG female mice
Wild animals	The study did not involved wild animals
Field-collected samples	The study did not involve samples collected from the field
Ethics oversight	All animal procedures and studies have been approved by the French ministère de l'éducation nationale de l'enseignement supérieur et de la recherche (APAFIS#2790-2015112015055793).

Note that full information on the approval of the study protocol must also be provided in the manuscript.

Human research participants

Policy information about studies involving human research participants

Population characteristics	At this interim cut-off, median age (interquartile range) for the study population was 65 years (55-71) with a higher proportion of men (60.1%) (Supplementary Table 1). The most common cancer types were non-small cell lung cancer (NSCLC) (n=142) followed by prostate (n=75), urothelial (n=30), gastrointestinal (n=17), gynecological (n=13) and breast cancers (n=8). Patients with less frequent tumor types were also included.<br>Regarding the last cancer treatment received at the time of inclusion, 127 patients (42%) had experienced disease progression

	with targeted therapies, 101 (33%) with immunotherapy and 75 (25%) with anti-androgen therapy (Figure 3).
Recruitment	Patients must have had either an initial response, defined as partial response (PR) or complete response (CR) by RECIST 1.1, or a stable response (SD) for at least 24 weeks, and develop disease progression while actively receiving molecular targeted therapy or immunotherapy. Key eligibility criteria for study entry are summarized in Table 1. Patients are included during a consultation with their referring physician and sign the informed consent.
Ethics oversight	The MATCH-R trial was approved by the ethics committee at Institut Gustave Roussy, the French National Medicines Safety Agency (ANSM), and was done in accordance with the Declaration of Helsinki.

Note that full information on the approval of the study protocol must also be provided in the manuscript.

Clinical data

Policy information about clinical studies

All manuscripts should comply with the ICMJE guidelines for publication of clinical research and a completed CONSORT checklist must be included with all submissions.

Clinical trial registration	NCT02517892
Study protocol	https://clinicaltrials.gov/ct2/show/NCT02517892
Data collection	Demographic and clinical data are prospectively collected together with pathology records using an electronic CRF with Macro software
Outcomes	In order to study the evolution of clonal architecture of tumors from patients treated with molecular targeted agents the outcomes are:<br>- Type and Frequency of molecular alterations in resistant tumors<br>- Comparison of molecular profile in patients pre-treatment and at progression with molecular targeted therapies<br>These measures are assessed using electronic CRF and SAS software v9.4
